# Overcoming Resistance to Immunotherapy in Advanced Cutaneous Squamous Cell Carcinoma

**DOI:** 10.3390/cancers13205134

**Published:** 2021-10-13

**Authors:** Natalia García-Sancha, Roberto Corchado-Cobos, Lorena Bellido-Hernández, Concepción Román-Curto, Esther Cardeñoso-Álvarez, Jesús Pérez-Losada, Alberto Orfao, Javier Cañueto

**Affiliations:** 1 IBMCC-CSIC, Laboratory 7, Campus Miguel de Unamuno s/n, 37007 Salamanca, Spain; nataliagarciasancha@usal.es (N.G.-S.); rober.corchado@usal.es (R.C.-C.); jperezlosada@usal.es (J.P.-L.); 2 Instituto de Investigación Biomédica de Salamanca (IBSAL), Hospital Universitario de Salamanca, Paseo de San Vicente 58-182, 37007 Salamanca, Spain; lbellido@saludcastillayleon.es (L.B.-H.); cromancurto@gmail.com (C.R.-C.); orfao@usal.es (A.O.); 3 Departament of Medical Oncology, Hospital Universitario de Salamanca, Paseo de San Vicente 58-182, 37007 Salamanca, Spain; 4 Departamento de Dermatología, Hospital Universitario de Salamanca, Paseo de San Vicente 58-182, 37007 Salamanca, Spain; mesthercardenoso@gmail.com; 5 IBMCC-CSIC, Laboratory 11, Campus Miguel de Unamuno s/n, 37007 Salamanca, Spain; 6 Cytometry Service (NUCLEUS) and Department of Medicine, University of Salamanca, 37007 Salamanca, Spain; 7 Centro de Investigación Biomédica en Red de Cáncer (CIBERONC) (CB16/12/00400, CB16/12/00233, CB16/12/00369, CB16/12/00489 and CB16/12/00480), Instituto Carlos III, 28029 Madrid, Spain

**Keywords:** cutaneous squamous cell carcinoma, immunotherapy, anti-PD1, biomarkers, predictive medicine, personalized medicine, cancer, immune system

## Abstract

**Simple Summary:**

Cutaneous squamous cell carcinoma (CSCC) is the second most frequent cancer in humans. The therapeutic landscape of CSCC has change in recent years, after the approval of immune checkpoint inhibitors (ICI) in advanced CSCC. However, not all patients will respond to ICI, and those who respond may develop resistance over time. Understanding the predictors of response to immunotherapy and the mechanisms underlying primary and acquired resistance to ICIs may help identify which patients could best benefit from these therapies. Many treatment strategies are under development to overcome resistance to immunotherapy, such as immune checkpoint inhibitors plus vaccines, oncolytic virus, radiotherapy, chemotherapy, or tumor microenvironment modulators.

**Abstract:**

Cutaneous squamous cell carcinoma (CSCC) is the second most frequent cancer in humans, and is now responsible for as many deaths as melanoma. Immunotherapy has changed the therapeutic landscape of advanced CSCC after the FDA approval of anti-PD1 molecules for the treatment of locally advanced and metastatic CSCC. However, roughly 50% of patients will not respond to this systemic treatment and even those who do respond can develop resistance over time. The etiologies of primary and secondary resistance to immunotherapy involve changes in the neoplastic cells and the tumor microenvironment. Indirect modulation of immune system activation with new therapies, such as vaccines, oncolytic viruses, and new immunotherapeutic agents, and direct modulation of tumor immunogenicity using other systemic treatments or radiotherapy are now under evaluation in combined regimens. The identification of predictors of response is an important area of research. In this review, we focus on the features associated with the response to immunotherapy, and the evaluation of combination treatments and new molecules, a more thorough knowledge of which is likely to improve the survival of patients with advanced CSCC.

## 1. Introduction

Cutaneous squamous cell carcinoma (CSCC) is the second most frequent cancer in humans, with an estimated annual incidence of one million cases in the US and the cause of as many as 9000 deaths each year [1,2]. Its incidence is increasing by 3–8% per year in most countries [3] and, by 2030, the rate in Europe is expected to have doubled [4]. Although CSCC generally exhibits a benign clinical behavior, some cases may entail a poor prognosis. Local recurrence is estimated to occur in 5% of patients, lymph node metastasis in 3.7 to 5.8% and disease-specific death in 1.5 to 2.1% of cases [5,6]. CSCC is already a public health concern worldwide, and as life expectancy lengthens in general, it will become an even greater health problem. 

CSCC is especially common in elderly fair-skin men. It is associated with chronic sun exposure, and immunosuppression represents a major risk factor. Actinic keratosis is the most significant independent risk factor for CSCC development. Human papillomavirus infection [7], long-term scars, and inflammatory skin conditions are other well-known risk factors [8]. 

Ultraviolet exposure induces *P53* mutations and genomic instability. Consequently, mutations occur in tumor suppressor genes (such as *CDKN2A* and *NOTCH*) and oncogenes (such as *RAS*). The accumulation of mutations causes deregulation of relevant oncogenic pathways (EGFR overexpression and activation of MAPK and PI3K/mTOR pathways), which results in CSCC development. Epigenetic factors, such as the methylation status and the role of miRNAs, also contribute to CSCC development [9,10]. CSCC is the solid tumor with the highest mutational burden [11], which is part of the rationale that led to immunotherapy.

Immunotherapy has changed the therapeutic landscape of CSCC in recent years. Patients with locally advanced or metastatic CSCC who would not benefit from surgery are now candidates for immune checkpoint inhibitors (only two anti-PD1 drugs are currently FDA-approved) [12]. However, not all patients respond to immunotherapy, and some begin to respond but develop resistance over time. Reasons underlying this primary and acquired resistance to immunotherapy are a matter of intensive research [13,14]. It is also important to identify which patients would most benefit from these treatments, which is why research on biomarker signatures has become a priority. Finally, novel therapies to overcome resistance to immunotherapy and to increase the response rate and maintain remission once it has been achieved are being evaluated. Combinations of immune checkpoint inhibitors (ICIs) and of ICIs with other therapies (such as radiotherapy, chemotherapy and targeted therapy), together with cancer vaccines and oncolytic viruses make up the new treatment options under evaluation in clinical trials, many of which are yielding promising results [15]. 

In this review, we first describe the current evidence about immunotherapy in CSCC. We then summarize the predictors of response to immunotherapy. Finally, we discuss the state-of-the-art of the known mechanisms of resistance to immunotherapy and several therapies for overcoming resistance that are under investigation, paying particular attention to novel therapies in CSCC. 

## 2. Immunotherapy in Cutaneous Squamous Cell Carcinoma

### 2.1. Immune Checkpoint Inhibitors in Cutaneous Squamous Cell Carcinoma

#### 2.1.1. Cancer Immunotherapy and Tumor Immunology

Immunotherapy has become an established mainstream treatment for in cancer and has improved the prognosis and survival of many patients, including those with hematological dyscrasias and solid malignances. Tumor cells produce neoantigens that are recognized and targeted by the immune system as foreign molecules, thereby preventing carcinogenesis. 

**Figure 1:**
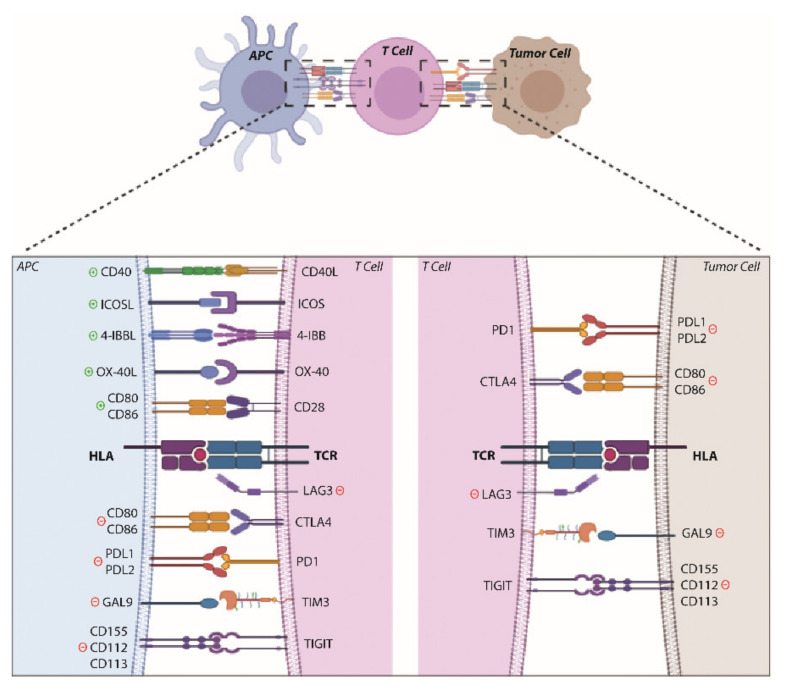
Scheme of co-stimulatory and co-inhibitory receptors implicated in the immune response. Created using BioRender.

Antigen-presenting cells (APCs) offer tumor neoantigens to the T-cell receptor (TCR) in naïve T cells through the major histocompatibility complex (MHC) (human leucocyte antigen, HLA). To complete T-cell activation, other co-stimulatory molecules are necessary. CD28 and B7 (CD80/CD86) are two such molecules that are required for full T-cell activation. However, co-inhibitory molecules that act as immune checkpoints are important for avoiding hyperstimulation and autoimmunity. For example, the CTLA-4 receptor is expressed in activated and regulatory T cells and competes with CD28 for B7, thereby preventing T-cell hyperactivation [16,17,18]. PD-1 also acts as a co-inhibitory receptor. It is expressed in T cells and binds to its ligand PD-L1, which is mainly expressed in tumor cells, thus preventing T-cell activation and inducing immunological exhaustion [19,20,21] (Figure 1). 

In this context, the immune system can recognize the tumor and fight against it (immunosurveillance). However, if this process is not successful, tumor cells may enter into an equilibrium phase, with incomplete tumor destruction and finally, the tumor may escape to immune control. This dynamic process is known as immunoediting. The cancer immunoediting hypothesis postulates a dual role of the immune system: first, it protects the host by eliminating tumor cells, and second, it promotes tumor development by selecting tumor variants with reduced immunogenicity [22,23].

One of the ways in which tumor cells actively evade their destruction by the immune system is expressing these molecules that inhibit T-cell activation and response. The study of these mechanisms has allowed important advances to develop antibodies against CTLA-4, PD-1 and PD-L1, which have revolutionized oncology in recent years. Seven immune checkpoints inhibitors have so far received FDA approval for use with different types of cancer: one CTLA-4 inhibitor (ipilimumab), three PD-1 inhibitors (nivolumab, pembrolizumab, and cemiplimab) and three PD-L1 inhibitors (atezolizumab, durvalumab, and avelumab). However, many molecules (e.g., 4-1BB, OX40, LAG3, ICOS) are involved in T-cell activation [24] (Figure 1), and other drugs against them are being evaluated and developed. 

#### 2.1.2. Immunotherapy in CSCC

CSCC exhibits the greatest tumor mutational burden, which results in higher levels of tumor neoantigens that may be targeted by the immune system [11]. Immunocompromised patients have a higher risk of developing CSCC because their immune system is less efficient detecting and destroying cancer cells [8]. Both these factors underpin the rationale for testing immunotherapy for CSCC.

The FDA (2018) and EMA (2019) approved cemiplimab (Libtayo) as the first immunotherapeutic drug for the treatment of locally advanced or metastatic CSCC in patients who are not candidates for curative surgery or radiotherapy [25,26]. Cemiplimab is a high-affinity human monoclonal antibody directed against PD-1. The robust responsiveness of CSCCs to cemiplimab was demonstrated in expanded phase 1 and phase 2 trials (NCT02383212 and NCT02760498). In these clinical trials, the response rates were between 41% and 53% and the rates of durable disease control were between 57% and 65%. The efficacy of the treatment of metastatic and of locally advanced cutaneous squamous cell carcinoma was similar [27,28,29,30]. The second anti-PD-1 approved by the FDA, in 2020, is pembrolizumab (Keytruda). This drug has been accepted for use in patients with recurrent or metastatic CSCC that cannot be cured with surgery or radiation [31]. Its antitumor activity and durable response were established in the KEYNOTE-629 and CARSKIN clinical trials (NCT03284424 and NCT02883556) in which the response rates were 34.3% and 42% and the disease control rates were 52.4% and 60%, respectively [32,33]. 

The other ICIs, such as nivolumab and ipilimumab, have also been studied in clinical trials and have proved their efficacy in monotherapy in some case reports [34,35,36]. The greatest advantages of immune checkpoint blockers have been impressive durable response rates and manageable treatment-related adverse events compared with conventional therapies [37].

### 2.2. Predictors of Response to Immunotherapy

**Figure 2:**
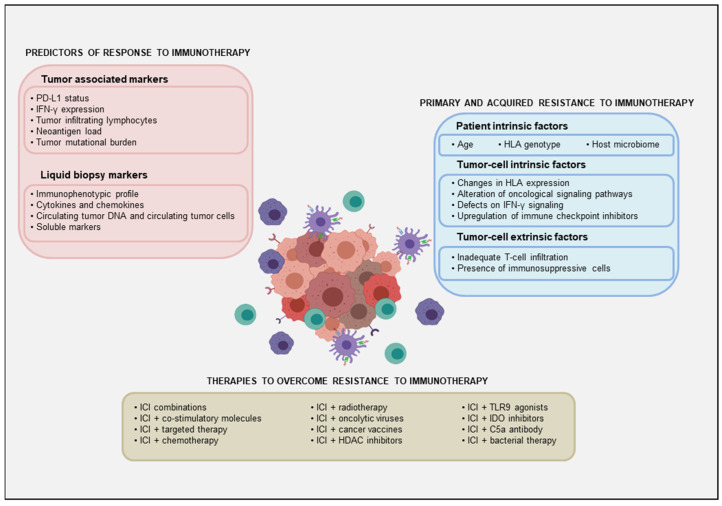
Representative scheme of the predictors of response to immunotherapy, the factors implicated in primary and acquired resistance to immunotherapy and the strategies to overcome these resistances.

About 50% of cancers will not respond to immunotherapy, so identifying predictors of response for checkpoint blockade-based immunotherapy has become a research priority. This will identify the patients who would respond best to the treatment, and thereby help maximize the therapeutic benefit. In recent years, numerous response predictors based on the gene expression status of the tumor (PD-L1 and IFN-γ expression), genomic changes (tumor mutational burden, T cell receptor clonality, neoantigen load and tumor aneuploidy) and immune cell infiltration have been found [38]. Biomarkers in peripheral blood are also being explored as non-invasive techniques (Table 1 and Figure 2).

#### 2.2.1. Tumor-Associated Markers

##### PD-L1 Status

High levels of PD-L1, detected by immunohistochemistry, are associated with the response to immunotherapy in melanoma, non-small cell lung carcinoma (NSCLC), renal cell carcinoma, colorectal carcinoma, and castration-resistant prostate cancer [39,40,41,42]. However, some studies have shown that the association with response varies over time and with the tumor type, and a sizable proportion of responses occur in PD-L1-low/negative tumors [43]. In CSCC, response to cemiplimab is independent of PD-L1 status, and durable disease control is similar in patients with <1% of PD-L1 expression and those with >50% PD-L1 expression [30]. Expression levels of PD-L1 are intratumorally heterogeneous and dynamic. The variety of antibody clones and platforms used the multiple scoring criteria and the variations in methodology make it difficult to interpret PD-L1 levels [44]. 

PD-L1 is one of the biomarkers currently approved for clinical use, but only to identify PD-L1 tumor expression in certain tumor types, specifically in: NSCLC for treatment with pembrolizumab, cemiplimab, atezolizumab or nivolumab in combination with ipilimumab; urothelial carcinoma and triple-negative breast cancer (TNBC) for treatment with pembrolizumab, cemiplimab or atezolizumab; and gastric adenocarcinoma, cervical cancer, head and neck squamous cell carcinoma (HNSCC) and esophageal squamous cell carcinoma for treatment with pembrolizumab or cemiplimab [45]. 

##### Interferon-Gamma Expression

PD-L1 expression may be upregulated via interferon-gamma (IFN-γ). IFN-γ produced from CD8+ T cells drives IL-12 production by tumor-infiltrating dendritic cells, which are necessary to a successful anti-PD-1 therapy [46]. In NSCLC and melanoma, patients with a high level of mRNA expression of *IFNG* (the gene that encodes IFN-γ) exhibit longer progression-free and overall survival and have higher disease control rates with anti-PD-1 therapies [47]. In several solid tumors, responders and non-responders to pembrolizumab can be distinguished on the basis of the different levels of expression of genes associated with IFN-γ [48]. The 18-gene IFN-γ characterized by this group is better than PD-L1 immunohistochemistry at identifying patients who will respond to immunotherapy [48]. However, more experiments, currently being carried out [49,50], are needed to make clinical implementation possible. 

##### Tumor Mutational Burden

A high tumor mutational burden (TMB) is also associated with response to ICI and improved overall survival in melanoma [51,52], NSCLC [53], urothelial carcinoma [54], among other cancers [55,56]. CSCC displays the greatest tumor mutational burden, and a large TMB has been linked to a good clinical response to immunotherapy [11,57,58]. Nevertheless, some patients with a large TMB may not respond to ICI therapy [30,59]. In some tumors, such as glioma, TMB is associated with shorter overall survival [60,61]. One advantage of TMB is that it can predict responses to CTLA-4 antibodies and PD-1/PD-1 inhibitors, but measuring TMB by whole exome sequencing or by sequencing 300–400-gene panels is an expensive and not routinely available option. Moreover, it is difficult to establish a threshold for all cancer types, hampering standardization of the technique [60,62]. Nonetheless, FDA approved FoundationOne CDx to identify patients with unresectable or metastatic solid tumors with a high mutational burden (≥10 mutations/megabase) for whom treatment with pembrolizumab may be appropriate [63]. 

##### Neoantigen Load

High TMB increases the capacity of the tumor to generate new neoantigens. Tumors loaded with more neoantigens are more likely to respond to immunotherapy [64,65,66]. Knowledge of the neoantigen landscape, derived from proteomic experiments and computational predictive algorithms [43,67], may enable us to adopt a precision-medicine approach, although the technology required is complex.

##### Tumor-Infiltrating Lymphocytes

Tumor-infiltrating lymphocytes (TILs) play an important role in the response to immunotherapy. TILs comprise primarily CD8+ cytotoxic T cells and CD4+ helper T cells, including regulatory T cells (Tregs), which are exemplified by the expression of FOXP3 and CD25. TILs also encompass a smaller proportion of B and natural killer cells. In melanoma, preexisting CD8+ T cells at the invasive front (the edge of the tumor) are essential for tumor regression following pembrolizumab therapy [68]. In melanoma, patients treated with PD-1 antibodies have a response rate of 78.6% when pretreatment tumor biopsies contain more than 20% of tumor-infiltrating CD8+ T cells that express high levels of PD-1 and CTLA-4, in contrast to non-responders, who feature fewer than 20% of these cells and a 0% response rate [69]. In metastatic NSCLC and melanoma treated with pembrolizumab or nivolumab, the response rates are low (13.3 and 0%, respectively) when the pretreatment CD8+/CD4+ TIL ratio is less than 2, whereas they are high (50.0 and 81.3%, respectively) when the ratio is greater than 2 in NSCLC and greater than 2.7 in melanoma [70]. The customary evaluation of TILs using hematoxylin-eosin and immunohistochemistry has revealed notable inter- and intra-observer variability. New tools based on flow cytometry, RNA-sequencing and digitalization of images are being developed to validate and promote an immunoscore-based method [71,72]. 

#### 2.2.2. Liquid Biopsy Markers

Most of the data on prediction of response to immunotherapy have focused on tumor features. Nevertheless, tumors are sometimes less accessible, and the role of the host immune system is a critical consideration. Determining the host immunological profile in blood samples allows assessment of the tumor immunovigilance state, the risk of tumor progression, and the response to treatment, which can help in establishing a panel of biomarkers that predict response. 

##### Immunophenotypic Profile

We currently know little about the immunological profile of patients receiving treatment with cemiplimab and pembrolizumab in CSCC, and most of the information available comes from studies in melanoma. In this disease, some baseline laboratory markers have been linked to the response to ipilimumab (such as high levels of CD4+ and CD8+ T lymphocytes [73], low levels of neutrophils and LDH [74], myeloid and monocyte precursors [75]) and to the response to pembrolizumab (such as high eosinophil levels, low LDH levels, and high total lymphocyte count [76]). Furthermore, changes in the immune profile during treatment have implications for the prognosis of the disease, such as a reduction in Treg/FoxP3+ lymphocyte levels and an increase in the overall lymphocyte count [77], or an increase in the total lymphocyte and eosinophil counts [78]. 

##### Cytokines and Chemokines

The profile of peripheral blood cytokines and chemokines, which is related to the immune cell populations, offers an opportunity to define the prognosis of the disease. The level of expression of certain cytokines is known to be associated with better responses [79]. In melanoma, high levels of IL-6 reduce the likelihood of response to ipilimumab [80]. In melanoma and NSCLC patients, an early decrease in IL-8 is associated with the best response to nivolumab and pembrolizumab [81]. IL-8 is a powerful chemoattractant for neutrophils and other immune-suppressive cells and elevated baseline levels of serum IL-8 correlate with reduced clinical benefit of ICI in different advancer cancer [82]. 

##### Circulating Tumor DNA and Circulating Tumor Cells

Circulating tumor DNA (ctDNA) is one of the most reliable biomarkers available in liquid biopsy. Low basal levels of ctDNA are correlated with good prognosis and best clinical response in melanoma [83,84] and other solid tumors [85]. The TMB can be measured in ctDNA [86] and the FDA recently approved FoundationOne Liquid CDx and Guardant360 CDx [45] for comprehensive tumor mutation profiling through liquid biopsy sampling. Patients with high levels of blood-based TMB respond better to ICIs [87,88], although this is not well established for all types of cancer; the concordance of blood-TMB and tissue-TMB is currently being examined. 

Circulating tumor cells (CTCs) also identify responders and non-responders. A reduction in CTC frequency during pembrolizumab or ipilimumab treatment improves progression-free survival and high quantities of CTCs are related to a higher risk of relapse [89]. In CTCs, PD-L1 expression can be determined and patients with CTCs/PD-L1+ have better progression-free survival than CTCs/PD-L1- patients when they receive pembrolizumab [90]. 

##### Soluble Markers 

Soluble forms of many immune regulatory molecules, both co-stimulatory and co-inhibitory molecules, including sCTLA4 and sPD-L1, are detected in plasma of cancer patients. Higher sPD-L1 plasma levels are associated with poor prognosis in melanoma [91] and with lower nivolumab efficacy in NSCLC [92].

The combination of biomarkers may have greater predictive power than the individual markers [93,94]. A recent meta-analysis published reveals a model that combines 11 factors to predict sensitization to ICI. The multivariable model includes clonal, frameshift insertion/deletion and nonsense-mediated decay-escaping TMB, signatures associated with tobacco, UV, APOBEC and T cell-related inflammation, sex, and gene expression values for CD8A, CXCL9, and PD-L1, with better predictive value than one factor alone [95]. An integrated approach with new bioinformatic tools can help us stratify patients and select the best treatment. This will tell us which patients will, or will not, respond to ICI monotherapy. Some of those who do not respond may benefit from new therapies that are being developed to overcome resistance to immunotherapy. We discuss these therapies below. 

## 3. Mechanisms of Resistance to Immunotherapy 

Despite the success of immune checkpoint inhibitors, some patients treated with ICIs do not benefit from treatment (primary resistance), and some of those who initially do, become resistant over time (acquired resistance) (Table 2 and Figure 2). Primary and acquired resistance are both a result of complex and constant interactions between cancer cells and the tumor microenvironment. Understanding the mechanisms by which this resistance occurs is essential for developing strategies to overcome resistance. 

### 3.1. Primary Resistance to Immune Checkpoint Inhibitors

In primary resistance, patients do not respond at all to ICIs, facilitating the progress of the disease. The response rate to single-agent immune checkpoint blockade ranges from 40 to 70% in different types of cancer. Patient-intrinsic factors (such as age, HLA genotype and gut microbiome), tumor cell-intrinsic factors (such as insufficient tumor antigenicity, loss of HLA expression and alterations of several signaling pathways) and tumor cell-extrinsic factors (such as changes in tumor-associated stroma) are involved in primary resistance to immunotherapy [13,14,137]. 

#### 3.1.1. Patient-Intrinsic Factors

##### Immunosenescence

As patients age, their immune system function becomes increasingly limited. This process, known as immunosenescence, is characterized by significant effects upon innate and adaptive immune responses. 

With respect to innate immunity, aging produces changes in monocytes and macrophages (reduced phagocytic activity, HLA II expression and ROS production), dendritic cells (slower maturation and reduced antigen presentation, defective TLR expression and signaling) and neutrophils (reduced chemotaxis and altered TLR expression). 

The adaptive response is limited by a drop in the frequencies of naïve B and T cells and a rise in those of senescent and exhausted T cells, Treg and myeloid-derived suppressor cells (MDSCs) [96,97,98]. All these changes compromise clonal expansion and cytokine and antibody production, weakening the immune response. 

The results of clinical trials in this area are variable. The elderly group is underrepresented because their co-morbidities are sometimes exclusion criteria. However, in patients older than 75 years, resistance to anti-PD-1/anti-PD-L1 therapy has been observed in squamous cell carcinoma and adenocarcinoma of the lung, renal cell carcinoma and squamous cell carcinoma of the digestive tract. Nevertheless, two other studies in NSCLC reported the same benefit in the elderly as that seen in younger individuals [99,100]. 

##### HLA Haplotypes

The human leukocyte antigen class I (HLA-I) genotype is linked to differential immune responses, including different responses to ICIs. Homozygosity in at least one HLA-I locus in patients treated with anti-CTLA-4, anti-PD-1, anti-PD-L1 or with a combination of ICIs for different types of cancer (mostly melanoma and NSCLC) is associated with shorter overall survival. Conversely, maximal heterozygosity at HLA-I loci with a high TMB is associated with extended survival after ICI treatment [101]. Moreover, HLA-I genotype with two alleles with more divergent sequences, measured as HLA-I evolutionary divergence (HED), enables presentation of more diverse immunopeptidomes and is correlated with better survival after treatment with ICIs [102]. 

##### Host Microbiome

Links between the host microbiome and the response to ICIs have emerged in recent years [103]. In melanoma, patients treated with anti-PD-1 with highly diverse and abundant Faecalibacterium have enhanced systemic and anti-tumor responses mediated by increased antigen presentation. In contrast, patients with low Bacteroidales diversity have impaired anti-tumor immune responses mediated by limited intratumoral lymphoid and myeloid infiltration and higher frequencies of Treg cells and MDSCs in blood [104]. Other bacterial species found to be more abundant in responders include Bifidobacterium longum, Collinsella aerofaciens, and Enterococcus faecium [105]. 

#### 3.1.2. Tumor-Associated Factors

##### Tumor Cell-Intrinsic Factors

Tumor cell-intrinsic factors are involved in primary resistance. The loss of HLA expression, the alteration in antigen processing machinery, the lack of antigenic mutations, the constitutive PD-L1 expression and the alteration in particular signaling pathways are the most significant tumor-intrinsic factors [137]. 

Tumor cells can avoid being attacked by T cells by downregulating HLA expression. An HLA-low phenotype has been observed in NSCLC, breast, prostate and colorectal cancers, HNSCC, hepatocellular carcinoma and melanoma. Several genes, such as *TAP1*, *TAP2*, *B2M*, *TAPBPR*, *ERAP1*, are involved in the synthesis, assembly, transport and surface expression of HLA I molecules, and defects in the HLA I pathways may result in the loss of 0 to 93% of HLA I expression in different types of cancer [106]. Losing HLA I antigen presentation machinery makes CD8 T cells unable to identify tumor cells, thereby making it possible for cancers to evade immune control. Loss of antigenicity is also associated with a loss of immunogenicity, due to low tumor mutational burden [107]. 

Alternation in oncological signaling pathways may result in resistance to ICIs. Abnormal expression of the mitogen-activated protein kinase (MAPK) pathway is associated with impaired recruitment and function of tumor infiltrate lymphocytes through expression of VEGF and other inhibitory cytokines [108,109]. In this context, it has been shown that melanomas become resistant to immunotherapy when they have previously acquired resistance to MAPK targeted therapy, in a process knowing as cross-resistance. It is due to a reactivated MAPK pathway and the induction of an immunosuppressive tumor microenvironment that lacks functional CD103+ dendritic cells, precluding an effective T cell response [110]. Similarly, loss of PTEN, which enhances PI3K signaling, is associated with resistance to immune checkpoint therapy [111]. The resistance due to PTEN deficiency is associated with high levels of VEGFA and STAT3 [112], stronger PD-L1 expression [113] and lower CD8+ T-cell density [112]. Constitutive WNT/β-catenin expression reduces expression of the cytokine CCL4 necessary to recruit CD103+ dendritic cells, which are involved in T-cell priming [114]. The occurrence of somatic JAK1/2 mutations in cancer cells leads to loss of IFN-γ signaling, making it another mechanism producing primary resistance to PD-1 blockade therapy [115]. 

##### Tumor Cell Extrinsic Factors

Tumor cells do not work alone but in conjunction with their environment, interacting with the extracellular matrix within the stroma and with the immune cells of the tumor microenvironment. The absence of T cells near the tumor, the presence of immunosuppressive cells and the expression of different inhibitory immune checkpoints have all been implicated in primary resistance.

Inadequate T-cell infiltration may be due to a variety of factors such as poor immunogenicity, downregulation of chemokines required for T-cell recruitment (CXCR3, CXCL9, CXCL10) by epigenetic silencing and upregulation of the endothelin B receptor or VEGF overexpression [116]. T-cell function may be hindered by the presence of immunosuppressive cells in the tumor microenvironment. Tregs are known to suppress effector T-cell responses by secreting certain inhibitory cytokines such as IL-10, IL-35 and TGF-B, or by direct cell contact [117]. Greater infiltration of Tregs in the tumor is correlated with poor prognosis [118] and primary resistance to anti-PD-1 therapy [119]. MDSCs, a group of immature myeloid cells with suppressive competence in the tumor microenvironment, have been implicated in angiogenesis, tumor cell invasion, and metastasis [120]. Accumulation of circulating MDSCs is negatively associated with ICI efficacy [75,121,122] and eradicating them could enhance clinical responses to immunotherapy. Tumor-associated macrophages (TAMs) also suppress T-cell activation and promote angiogenesis, contributing to immunotherapy resistance by overexpressing PD-1/PD-L1, TGF-β, VEGF, EGF, and MMP [123,124]. All these immune cells can express other co-inhibitors such as TIM-3, CTLA-4 and TIGHT to mediate tumor immune resistance. Moreover, peritumoral fibroblast that express TGFβ are also implicated in poor response and resistance to atezolizumab prohibiting infiltration of effector CD8+ T cells into the tumor parenchyma [125].

### 3.2. Acquired Resistance to Immune Checkpoint Inhibitors

Numerous patients respond to immunotherapy but develop resistance over time. For example, in melanoma patients treated with ipilimumab and nivolumab, 38% of those who responded developed resistance [138]. In patients with NSCLC who were treated with nivolumab, up to 65% of responders progressed after 4 years of follow-up [139]. Across tumor types, there is an inverse correlation between overall response rate to PD-1 blockade and the frequency of acquired resistance [140]. Mechanisms of acquired resistance also lead to changes in HLA expression, altered IFN-γ signaling and poor neoantigen recognition [140].

Defective HLA class I antigen processing due to mutations in β2-microglobulin (B2M), which is required for HLA class I folding and transport to the cell surface [126,127], has been observed in patients with melanoma [128,129], lung cancer [130] and mismatch repair-deficient tumors [131] whose tumor initially regressed in response to ICIs but whose disease progressed some years later. Alterations in the IFN-γ pathway have also been implicated in the loss of HLA class I [128]. Defects in the IFN-γ pathway are produced by inactivating mutations in Janus kinases (*JAK1* or *JAK2*) or in interferon-gamma receptor 1 (*IFNGR1*) [128,132]. Lack of IFN responsiveness also results in the loss of PD-L1 expression [128]. Dysfunctional tumor antigen-presenting machinery reduces tumor visibility, leading to acquired ICI resistance. Tumor recognition can also be hampered by the loss of somatic mutations encoding tumor neoantigens through clonal selection, epigenetic repression or copy-number loss, leading to immune evasion and clinical progression [133]. In NSCLC, tumors with acquired immunotherapy resistance show genomic changes in genes encoding tumor neoantigens that can be recognized by T cells [134].

Additional changes known to influence acquired resistance are the upregulation of other T-cell checkpoints (TIM3 and LAG) [135], the loss of PTEN and the increase in WNT-β-catenin activity, which is linked to the promotion of Treg and changes in the priming of dendritic cells [136].

## 4. Overcoming Resistance to Immune Checkpoint Inhibitors

To overcome the resistance to ICIs, it is necessary to enhance the anti-tumor activity of the immune system. Combined treatment regimens and new therapies based upon synergistic effects of targeting different immune escape pathways are emerging (Figure 2). The therapies to overcome immunotherapy resistance in CSCC currently being studied are summarized in Table 3.

### 4.1. ICI Combinations

One of the first strategies used to bypass resistance is the use of a combination of immune checkpoint inhibitors. Anti-CTLA-4 (ipilimumab) plus anti-PD-1 (nivolumab) treatments are combinations approved for the treatment of melanoma [141,142], renal cell carcinoma [143,144], colorectal cancer [145], non-small cell lung cancer [146], hepatocellular carcinoma [147] and pleural mesothelioma [148]. The regulatory roles of CTLA-4 and PD-1 pathways are distinct, and simultaneously blocking the two receptors produces a synergistic effect [149,150].

In CSCC, ipilimumab is currently being tested in combination with nivolumab in a comparison with neo-adjuvant nivolumab monotherapy (NCT04620200), and combined with nivolumab and tacrolimus in treating kidney transplant recipients with metastatic CSCC (NCT03816332). However, combination therapy increases the incidence and severity of side effects. The median time to onset of a fatal adverse event tends to be earlier for a combination treatment than for monotherapy, and ICI-related deaths in combination therapies are attributed to colitis and myocarditis [151,152].

Numerous co-inhibitory molecules on the T-cell surface have been characterized in the context of T-cell activation [24]. LAG-3, TIM-3 and TIGIT are co-inhibitory molecules that regulate T-cell response and promote T-cell inhibition [153,154]. Resistance to PD-1 blockade has sometimes been associated with upregulation of these molecules [135], which has led to antibodies towards these molecules being developed and combined with traditional ICIs [155,156,157]. The combination of the anti-LAG-3 BMS-986016 (relatlimab) plus nivolumab strengthens the response in melanoma patients who are resistant to anti-PD-1/anti-PD-L1 therapy [158] (NCT01968109). Other anti-LAG3 agents, such as IMP-321 and LAG525, are under evaluation in a variety of cancer types [155] (NCT02676869, NCT03625323 and NCT03499899). Anti-Tim-3 and anti-TIGIT antibodies, in combination with anti-PD-1, have shown their efficacy in advanced cancers in mouse models [156,157,159]. The efficacy of these new drugs in CSCC has not yet been studied, but their combinations might be attractive options for fighting anti-PD1 resistance in this tumor.

### 4.2. Combination with Co-Stimulatory Molecules of T-Cell Response

OX40, ICOS and CD27 are co-stimulatory receptors present in T cells and natural killer cells that induce cellular activation. Specific agonist antibodies to these molecules have been developed to boost the immune response [160]. Anti-OX40 monotherapy suppressed tumor growth in preclinical models and enhanced anti-tumor T-cell activity when combined with ICIs [161]. In CSCC, triggering OX40 with an agonist antibody overcame the suppression exerted by Treg, increasing T-cell effector proliferation in vitro [162]. However, when the agonist BMS-986178 has been evaluated in patients with advanced cancer in monotherapy or in combination with nivolumab and/or ipilimumab (NCT02737475), no clear advantage was observed [163]. SL-279252, a bi-functional fusion protein that binds simultaneously to PD-L1 and OX-40 stimulating anti-tumor T-cell activity, is currently being tested in a clinical trial in several types of solid cancer, including CSCC (NCT03894618).

### 4.3. Combination with Chemotherapy

Although cancer chemotherapy has customarily been considered immunosuppressive, it is now accepted that certain cytotoxic agents can boost tumor immunity. Chemotherapy induces immunogenic cell death and changes in the tumor microenvironment. On the one hand, cytotoxic drugs attack cells, promoting their death. Dead cells release tumor antigens that bind to their receptors, activating the effector lymphocytes. Moreover, cytotoxic drugs abrogate Treg and MDSC activity, enhance dendritic cell activity, promote anti-tumor CD4+ T-cell phenotype and cell recognition [164]. FDA approved pembrolizumab in combination with chemotherapy (carboplatin and either paclitaxel or nab-paclitaxel) for treating metastatic squamous NSCLC [165] and nivolumab plus ipilimumab and chemotherapy (platinum) for metastatic NSCLC with no EGFR or ALK aberrations [166]. Recently, pembrolizumab plus paclitaxel or pembrolizumab plus gemcitabine and carboplatin have been approved for the treatment of recurrent inoperable or metastatic triple-negative breast cancer [167], and in HNSCC pembrolizumab in combination with platinum and 5-FU [168] (NCT02358031). However, these combinations have not yet been explored in the context of CSCC.

### 4.4. Combination with Radiotherapy

Radiotherapy is thought to function similarly to chemotherapy, inducing immunogenic cell death and increasing tumor antigens and damage-associated molecular patterns (DAMPs), which prompt antigen presentation activity and T-cell priming. Radiotherapy also enhances infiltration of CD4+, CD8+ T cells and cytotoxic NK into the tumor microenvironment [169]. The combination of radiotherapy and ICIs is being evaluated in different tumors types and stages, in preclinical settings and in clinical trials [170,171,172]. In CSCC, a case report showed complete remission in a patient treated concurrently with radiotherapy and pembrolizumab [173]. A clinical trial in patients with high-risk CSCC of the head and neck (NCT03057613), and another employing quad-shot palliative radiotherapy (NCT04454489), are underway. In the UNSCARRed study, avelumab, and radical radiotherapy are combined to treat unresectable CSCC (NCT03737721). When combining radiotherapy and immunotherapy, radiotherapy doses must be optimized. Otherwise, the radiation has an immunosuppressive effect [169].

### 4.5. Combination with Targeted Therapies

Combining anti-PD-L1/PD1 immunotherapy with targeted therapy could improve therapeutic outcomes. MYC overexpression, EGFR and KRAS mutations, PTEN deletions and MEK/ERK alterations are known to induce PD-L1 expression [174]. In melanoma, the combination of vemurafenib (BRAF inhibitor), cobimetinib (MEK inhibitor), and atezolizumab showed an objective response rate of 71.8% [175] and longer median progression-free survival [176]. In CSCC, EGFR overexpression is associated with poor prognosis [177]. The combinations of cetuximab, an EGFR inhibitor, with pembrolizumab (NCT03082534 and NCT03666325), and with avelumab (NCT03944941), other anti-PD-L1, are currently under evaluation. ASP-1929, an antibody conjugate of cetuximab and IRDye 700DX that can be photoactivated, is being combined with pembrolizumab or cemiplimab to treat recurrent/metastatic head and neck squamous cell carcinoma and locally advanced/metastatic CSCC with EGFR overexpression (NCT04305795). Cobimetinib, in combination with atezolizumab, is also being tested in CSCC (NCT03108131).

### 4.6. Combination with Oncolytic Viruses and Cancer Vaccines

Oncolytic viruses (OVs) are emerging as important biological agents in cancer treatment. Native or genetically modified, they have the ability to kill cancer cells and induce systemic anti-tumor immunity, transforming “cold” into “hot” tumors [178,179]. To date, one OV therapy has been approved by the FDA for treating advanced melanoma: talimogene laherparepvec (T-VEC), a modified herpes simplex virus (HSV) that includes a gene that codes for granulocyte macrophage colony-stimulating factor (GM-CSF) to enhance durable systemic anti-tumor immune responses [180,181]. Intralesional T-VEC has been associated with an increase in melanoma-specific CD8 T cells and a corresponding decrease in suppressive immune cells, such as CD4+ FoxP3+ regulatory T cells and MDSCs within the tumor microenvironment [182]. The combination of T-VEC with ipilimumab [183,184] or pembrolizumab [185] has been explored in melanoma too, revealing a response rate double that achieved with ICI monotherapy. In CSCC, T-VEC is currently tested in monotherapy (NCT03714828), in combination with nivolumab (NCT02978625) and with panitumumab, an EGFR antibody (NCT04163952). RP1 is another modified HSV, which encodes a fusogenic glycoprotein derived from gibbon ape leukemia virus (GALV-GP-R) protein and GM-CSF. The efficacy of RP1 is being tested in the context of CSCC in adult hepatic and renal transplant recipients delivered by intratumoral injection (NCT04349436) and in combination with cemiplimab or nivolumab in immunocompetent patients (NCT04050436 and NCT03767348). Two other modified HSV-1s have been tested in CSCC: HF10 (NCT01017185) and ONCR-177, alone and in combination with pembrolizumab (NCT04348916).

A wide range of viruses has been investigated to determine their potential value as cancer therapeutic agents. In addition to those of herpesvirus, modifications of adenoviruses, vaccinia viruses, measles viruses, coxsackieviruses, polioviruses, retroviruses, reoviruses, parvoviruses and vesicular stomatitis viruses have been examined and some are currently the subject of clinical trials [178,179,186].

Immune responses may also be boosted by methods involving cancer vaccines that are designed to induce or amplify pre-existing cellular and humoral immune responses against target tumor-associated antigens (TAAs) or tumor-specific antigens (TSAs). TAAs are self-antigens that are preferentially or abnormally expressed in tumor cells, although they may also be expressed in normal cells. TSAs comprise antigens expressed by oncoviruses and neoantigens encoded by cancer mutations and are characterized by high immunogenicity. The majority of neoantigens are unique to individual patients and can be detected by computational algorithms for the purpose of designing personalized therapies [187,188,189]. Several therapeutic vaccine strategies have been developed, including whole tumor cell-based vaccines, protein- and peptide-based vaccines, RNA and DNA vaccines, viral vectors engineered to express tumor antigens and dendritic cell-based vaccines [187,190]. In 2010, the FDA approved the clinical use of Sipuleucel-T, the first cancer vaccine for treating castration-resistant prostate cancer based on enriched ex vivo dendritic cells of each patient [191]. IFx Hu2.0, a whole-cell cancer vaccine, is currently under trial in monotherapy in Merkel cell carcinoma and CSCC (NCT04160065). CIMAvax, a recombinant human EGF-rP64K/montanide ISA 51 vaccine, is being tested in advanced CSCC of the head and neck and NSCLC in combination with nivolumab or pembrolizumab (NCT02955290). In CSCC and metastatic melanoma, Ad/MG1-MAGEA3 is currently being assayed alone or in combination with pembrolizumab (NCT03773744). This is an innovative strategy that combines cancer vaccination with oncolytic virotherapy. It involves two viruses —a replication-deficient adenovirus type 5 (Ad) and a modified Maraba virus as an oncolytic rhabdovirus (MG1)—expressing the same TMA (Melanoma-associated antigen 3, MAGEA3) [192].

### 4.7. Other Combinations

Supplementing immunotherapy with epigenetic modulators, such as histone deacetylase inhibitors (HDACis), may decrease tumor progression [193,194]. HDACis reduce the expression of various inflammatory cytokines (IL-6, IL-2, IL-10 and IFN-γ), enhance infiltration of immune cells, increase central and effector T-cell memory and reduce pro-tumorigenic M2 macrophages [195,196]. Currently, in CSCC, pembrolizumab is combined with abexinostat, an HDACi (NCT03590054).

Toll-like receptors (TLRs) are a family of molecules capable of recognizing pathogen-associated molecular patterns (PAMPs) and of inducing adaptive immune responses [197]. TLR agonists and antagonists have been designed to enhance immunity and are currently being clinically trialed in monotherapy and in combination with anti-PD-1 therapy [198]. The TLR9 agonist cavrotolimod (AST-008) is being tested in combination with pembrolizumab or cemiplimab in Merkel cell carcinoma, CSCC and melanoma (NCT03684785).

Indoleamine-2,3-dioxygenase (IDO) is an enzyme that lowers the level of tryptophan, induces cell-cycle arrest and effector T-cell apoptosis, and promotes Treg activity [199]. The presence of IDO in the tumor microenvironment is considered a possible mechanism of resistance to immunotherapy and IDO inhibitors (epacadostat and indoximod) have been combined with ipilimumab, nivolumab, or pembrolizumab in melanoma [200], but not so far in CSCC.

Levels of TAM and MDSCs can be reduced using colony-stimulating factor 1 receptor (CSF1R) inhibitors. For example, CSF1R blockade combined with anti-PD-1 or anti-CTLA-1 treatment is associated with enhanced tumor regression in a mouse model of pancreatic ductal adenocarcinoma [201]. In melanoma, numerous clinical trials are underway that combine antagonists of CSF1R or M-CSF, or GM-CSF agonists with ICI [202].

C5a is a potent anaphylatoxin that modulates inflammation, tumor formation and progression by suppressing the anti-tumor CD8+T-cell-mediated response and immunosuppression by recruiting MDSCs [203]. C5a antibody (vilobelimab/IFX-1) is currently tested alone or in combination with pembrolizumab in locally advanced or metastatic CSCC (NCT04812535).

Finally, since the gut microbiome has been implicated in resistance to ICIs, combined therapies with bacteria plus immunotherapy have been developed. In mice with melanoma, a combination regimen of orally administered Bifidobacterium and anti-PD-L1 therapy abolishes tumor outgrowth [204]. Bifidobacterium species, being immunomodulators of the immune response, increase the infiltration of CD8+ effector T cells and enhance the production of IFN-γ. Moreover, the microbiota composition could predict the efficacy of immunotherapy agents (see above) [205]. A better understanding of the role of the microbiome will open up new avenues for developing new therapies [206].

## 5. Conclusions

The therapeutic landscape of cutaneous squamous cell carcinoma has changed since the approval of anti-PD-1 therapies. However, not all patients respond, and those who do can develop resistance over time. Therefore, it is important to develop good predictors of response to immunotherapy to be able to identify which patients could benefit from it, and to investigate new treatment regimens for overcoming immunotherapy resistance.

## Figures and Tables

**Table 1:** Predictors of response to immunotherapy.

Category	Predictors	Correlation	Advantages (and Approved Tests by FDA)	Disadvantages	References
**Tumor-associated markers**	**PD-L1 status**	High levels of PD-L1 are correlated with response to anti-PD-L1/PD-1 inhibitors	Immunohistochemistry detection is easy, cheap and automated Approved in NSCLC to treat with pembrolizumab, cemiplimab, atezolizumab or nivolumab in combination with ipilimumab Approved in urothelial carcinoma to treat with pembrolizumab, cemiplimab or atezolizumab Approved in triple-negative breast cancer to treat with pembrolizumab, cemiplimab, or atezolizumab Approved in gastric carcinoma, cervical cancer, HNSCC and ESCC to treat with pembrolizumab or cemiplimab	PD-L1-negative tumors also respond to anti-PD-L1 therapy PD-L1 expression is intratumorally heterogeneous and dynamic Different antibody clones and platforms used Multiple score criteria Methodological variabilities	[39,40,41,42,43,44,45]
	**IFN-y expression**	High levels of IFN-y expression are correlated with response to anti-PD-1 therapies	Higher capacity to detect patients who will respond to immunotherapy than PD-L1 immunohistochemistry	No standardized commercially available gene panel Expensive	[46,47,48,49,50]
	**Tumor mutational burden**	High levels of TMB are correlated with response to anti-CTLA-4 and anti PD-1/PD-L1 therapy (except glioma)	Applicable to most solid tumors and anti-CTLA4, anti-PD-L1 and anti-PD-1 therapies Approved for treating high-TMB solid tumors with pembrolizumab	Low-TMB tumors also respond to immunotherapy Whole-exome sequencing or sequencing of 300–400 genes panels is expensive Difficult to establish a threshold for all types of cancer	[51,52,53,54,55,56,57,58,59,60,61,62,63]
	**Neoantigen load**	High levels of neoantigen load are correlated with response to immunotherapy	Knowledge of the landscape of neoantigens to use a precision medicine approach	Complex technology High mutation load is not always correlated with response	[64,65,66,67]
	**Tumor-infiltrating lymphocytes**	High levels of CD8+ T cells, high ratio of CD8+/CD4+ T cells and high levels of CD8+/PD-L1+/CTLA-4+ lymphocytes are correlated with response to pembrolizumab and nivolumab	Easily detected by immunohistochemistry or hematoxylin-eosin staining	Inter- and intra-observer variability in hematoxylin-eosin and immunohistochemistry samples Score criteria not validated	[68,69,70,71,72]
**Liquid biopsy markers**	**Immunophenotypic profile**	High levels of CD4+ and CD8+ T lymphocytes and low levels of neutrophils, myeloid and monocyte precursor and Treg/FoxP3+ lymphocytes are correlated with response to ipilimumab High levels of eosinophils and high total lymphocyte count are correlated with response to pembrolizumab Low levels of LDH are correlated with response to ipilimumab and pembrolizumab	Ease of sample collection, non-invasive Possibility of collecting samples at different times during treatment Cheap	Not validated in clinical practice	[73,74,75,76,77,78]
	**Cytokines and chemokines**	High levels of IL-6 reduce the probability of responding to ipilimumab Early decrease in IL-8 is associated with best response to nivolumab or pembrolizumab	Ease of sample collection, non-invasive Possibility of collecting samples at different times during treatment Cheap	Not validated in clinical practice	[79,80,81,82]
	**Circulating tumor DNA and circulating tumor cells**	Low basal levels of ctDNA are correlated with good prognosis and best clinical response to immunotherapy High blood-based TMB measured in circulating tumor DNA are correlated with response to ICIs A reduction of circulating tumor cells improves progression-free survival during pembrolizumab or ipilimumab treatment Patients with CTCs/PD-L1+ have better progression-free survival when receiving pembrolizumab	Ease of sample collection, non-invasive Possibility of collecting samples at different times during treatment Cheap Test approved to measure TMB in liquid biopsy samples	Not validated in clinical practice	[45,83,84,85,86,87,88,89,90]
	**Soluble markers**	Higher sPD-L1 plasma level is associated with poor prognosis and lower nivolumab efficacy	Ease of sample collection, non-invasive Possibility of collecting samples at different times during treatment Cheap	Not validated in clinical practice	[91,92]

**Table 2:** Mechanisms of resistance to immunotherapy.

Type of resistance	Category	Factor	Relation	References
**Primary resistance to immunotherapy**	Patient-intrinsic factor	Immunosenescence	Aging limits immune response	[96,97,98,99,100]
		HLA genotype	Homozygosity in at least one HLA-I locus is associated with poor response to ICIs	[101,102]
		Host microbiome	Changes in diversity and abundance of host microbiome modify the response to ICIs	[103,104,105]
	Tumor cell-intrinsic factor	Downregulation of HLA expression	Loss of HLA-I expression reduces T-cell response	[106,107]
		Alteration of oncological signaling pathways	Abnormal expression of MAPK pathway, loss of PTEN, constitutive WNT/β-catenin expression, JAK1/2 mutations and loss of IFN-γ are involved in resistance to ICIs	[108,109,110,111,112,113,114,115]
	Tumor cell-extrinsic factor	Inadequate T-cell infiltration	Absence of T cells near the tumor reduces T cell response	[116]
		Presence of immunosuppressive cells	High level of infiltration of Treg, MDSCs and TAM suppress T-cell activation and is correlated with poor prognosis and resistance to ICIs	[117,118,119,120,121,122,123,124,125]
**Acquired resistance to immunotherapy**	Tumor cell-intrinsic factor	Changes in HLA expression	Mutations in β2-microglobulin are associated with acquired resistance to ICIs	[126,127,128,129,130,131]
		Defects of IFN-γ signaling	Escape mutations in IFN-γ pathway result in loss of HLA-I and PD-L1 expression and ICI resistance	[128,132]
		Mutations in genes that encode tumor neoantigens	Mutations in genes that encode tumor neoantigens reduce tumor recognition by immune system, leading to immune evasion and clinical progression	[133,134]
		Upregulation of other immune checkpoint receptors	Upregulation of TIM3 and LAG	[135]
		Alteration of oncological signaling pathways	Loss of PTEN and increase in WNT/β-catenin expression are linked to acquired resistance	[136]

**Table 3:** Combination therapies to overcome resistance to immunotherapy in cutaneous squamous cell carcinoma.

Type of combination	Drugs	Condition	NCT code
Combination of immune checkpoint inhibitors	Ipilimumab + nivolumab	In advanced CSCC prior to surgery	NCT04620200
	Ipilimumab + nivolumab + tacrolimus	Metastatic CSCC in treating kidney recipients	NCT03816332
Combination with co-stimulatory molecules	SL-279252 (binds to PD-L1 and OX-40)	Advanced CSCC	NCT03894618
Combination with chemotherapy	Currently not clinically trialed in CSCC		
Combination with radiotherapy	Pembrolizumab + radiotherapy (IMRT 60–66 Gy)	High risk CSCC of the head and neck	NCT03057613
	Pembrolizumab + quad-shot radiotherapy	Stage III and IV CSCC of the head and neck	NCT04454489
	Avelumab + radical radiotherapy	Unresectable CSCC	NCT03737721
Combination with targeted therapies	Pembrolizumab + cetuximab	Recurrent/metastatic CSCC	NCT03082534
	Pembrolizumab + cetuximab	Advanced/metastatic CSCC	NCT03666325
	Avelumab + cetuximab	Advanced/metastatic CSCC	NCT03944941
	Pembrolizumab/cemiplimab + ASP-1929 (EGFR antibody-dye conjugate)	Locally advanced or metastatic CSCC	NCT04305795
	Atezolizumab + cobimetinib	Metastatic CSCC	NCT03108131
Combination with oncolytic viruses	Nivolumab + talimogene laherparepvec	Advanced or refractory CSCC	NCT02978625
	Cemiplimab + RP1	Locally advanced or metastatic CSCC	NCT04050436
	Nivolumab + RP1	Locally advanced or metastatic CSCC	NCT03767348
	Pembrolizumab + ONCR-177	Advanced and/or refractory CSCC	NCT04348916
Combination with cancer vaccines	Nivolumab or pembrolizumab + CIMAVax vaccine	Stage III and IV CSCC of the head and neck	NCT02955290
	Pembrolizumab + Ad/MG1-MAGEA3	Previously treated CSCC	NCT03773744
Other combinations	Pembrolizumab + abexinostat (HDAC inhibitor)	Stage III and IV CSCC of the head and neck	NCT03590054
	Pembrolizumab or cemiplimab + cavrotolimod (TLR agonist)	Advanced/metastatic CSCC	NCT03684785
	Pembrolizumab + IFX-1 (C5a antibody)	Locally advanced or metastatic CSCC	NCT04812535
